# Comparing rates of mycobacterial clearance in sputum smear-negative and smear-positive adults living with HIV

**DOI:** 10.1186/s12879-021-06133-4

**Published:** 2021-05-22

**Authors:** Edith E. Machowski, Matebogo Letutu, Limakatso Lebina, Ziyaad Waja, Reginah Msandiwa, Minja Milovanovic, Bhavna G. Gordhan, Kennedy Otwombe, Sven O. Friedrich, Richard Chaisson, Andreas H. Diacon, Bavesh Kana, Neil Martinson

**Affiliations:** 1 Department of Science and Technology/National Research Foundation Centre of Excellence for Biomedical TB Research (CBTBR), University of the Witwatersrand, National Health Laboratory Service, Johannesburg, South Africa; 2 Perinatal HIV Research Unit (PHRU), SAMRC Soweto Matlosana Collaborating Centre for HIV/AIDS and TB, Faculty of Health Sciences, University of the Witwatersrand, Johannesburg, South Africa; 3 School of Public Health, Faculty of Health Sciences, University of the Witwatersrand, Johannesburg, South Africa; 4 TASK Applied Science, Bellville, Cape Town, South Africa and Pulmonology, Faculty of Medicine and Health Sciences, Stellenbosch University, Tygerberg, South Africa; 5 Johns Hopkins University School of Medicine, Baltimore, MD USA

**Keywords:** Tuberculosis, HIV, Smear microscopy, Xpert MTB/RIF, MGIT, Culturability, Bacterial load

## Abstract

**Background:**

Pulmonary tuberculosis (TB) in people living with HIV (PLH) frequently presents as sputum smear-negative. However, clinical trials of TB in adults often use smear-positive individuals to ensure measurable bacterial responses following initiation of treatment, thereby excluding HIV-infected patients from trials.

**Methods:**

In this prospective case cohort study, 118 HIV-seropositive TB patients were assessed prior to initiation of standard four-drug TB therapy and at several time points through 35 days. Sputum bacillary load, as a marker of treatment response, was determined serially by: smear microscopy, Xpert MTB/RIF, liquid culture, and colony counts on agar medium.

**Results:**

By all four measures, patients who were baseline smear-positive had higher bacterial loads than those presenting as smear-negative, until day 35. However, most smear-negative PLH had significant bacillary load at enrolment and their mycobacteria were cleared more rapidly than smear-positive patients. Smear-negative patients’ decline in bacillary load, determined by colony counts, was linear to day 7 suggesting measurable bactericidal activity. Moreover, the decrease in bacterial counts was comparable to smear-positive individuals. Increasing cycle threshold values (Ct) on the Xpert assay in smear-positive patients to day 14 implied decreasing bacterial load.

**Conclusion:**

Our data suggest that smear-negative PLH can be included in clinical trials of novel treatment regimens as they contain sufficient viable bacteria, but allowances for late exclusions would have to be made in sample size estimations. We also show that increases in Ct in smear-positive patients to day 14 reflect treatment responses and the Xpert MTB/RIF assay could be used as biomarker for early treatment response.

**Supplementary Information:**

The online version contains supplementary material available at 10.1186/s12879-021-06133-4.

## Introduction

In high tuberculosis (TB) burden settings HIV-infection confers markedly higher risk of TB infection and TB disease [1]. In general, pulmonary TB in persons living with HIV (PLH) presents with less cavitation and lower bacillary loads in sputum [2–6] and is therefore more likely to be below the detection limit of smear microscopy than in immunocompetent individuals [7]. Inclusion criteria for clinical trials of novel agents or regimens against TB often require smear-positive disease to assure baseline positive *Mycobacterium tuberculosis* cultures in samples taken prior to initiation of TB treatment to ensure measurable treatment responses from a high baseline [8, 9]. Smear-negative PLH are therefore underrepresented in studies of novel treatments for pulmonary TB [10–13].

Four semi-quantitative methods are used to infer bacillary load - the concentration of mycobacteria - in a biological sample [14–20]. Smear grading on Ziehl-Nielsen or auramine stained smears directly detects bacteria by light or fluorescent microscopy [14]. The Xpert MTB/RIF assay (Cepheid Innovation, Sunnyvale, CA) is based on nucleic acid amplification technology (NAAT) [15]. It has a reported limit of detection (LOD) of as low as 112.6 CFU/ ml (colony forming units per millilitre) and is increasingly replacing smear microscopy as the primary diagnostic measure on sputum from individuals with presumptive pulmonary TB [16–18]. Subsequently, the Xpert MTB/RIF-Ultra assay has become available that has a reported LOD of 15.6 CFU/ml [19]. The Xpert’s cycle threshold (Ct or Cq) reflects bacillary load as an inverse measure, where low values represent high bacillary numbers. The fluorescent signal produced from the NAAT emerges sooner if more DNA is present and in practice, those with *M. tuberculosis* detected results have Ct values from 15 to 28. In the automated Mycobacterial Growth Indicator Tube (MGIT) liquid culture system (Becton, Dickinson, Franklin Lakes, NJ), the time-to-positive (TTP) signal occurs sooner when bacillary load is high [20]. Positive results are detected from as low as 3 days with high loads but the system can flag positive to up to 42 days, after which it is considered negative. Bacterial cell counts on solid media provide numeric read outs and viable bacillary load can be quantified accurately. Cultures - although providing evidence of mycobacterial viability, which smear microscopy and Xpert are unable to do - may not be of immediate value because positive results for bacterial cell counts take at least 3 weeks for bacteria to form colonies, and MGIT culture can take up to 42 days for a positive result [14] by which time decisions about initiating TB treatment likely have already been made. Withholding treatment in patients potentially eligible for a clinical trial for the culture results would be unacceptable.

In this study we followed a cohort of adult PLH with pulmonary TB, both smear-negative and smear-positive, and subjected their serial sputum specimens whilst on therapy to these four semi-quantitative methods to characterise their initial, and extended mycobacterial responses to TB treatment. Our overall objective was to assess if HIV-infected, smear-negative patients with pulmonary TB have demonstrable bacillary responses to therapy and could be included in clinical trials of novel treatment regimens.

## Methods

### Study design

This was a three site, prospective case cohort study, in which we purposively recruited HIV-infected adult TB patients irrespective of their antiretroviral therapy status. After obtaining informed consent, adult patients with an initial public sector Xpert positive result were recruited at Soweto, Klerksdorp and Cape Town - into two studies with almost identical design. Patients were included if they had not taken TB medications in the 1 year prior to enrolment and were eligible for standard four-drug first-line TB regimen for the current TB disease episode. Patients were excluded if they had received more than 5 days of TB treatment before the first study sputum was taken, and if they had received any antibiotics with activity against *M. tuberculosis* in the previous 10 days (e.g. aminoglycosides, fluoroquinolones, etc.). Standard four-drug anti-TB therapy was initiated as soon as possible after: 2 months of isoniazid, rifampicin, pyrazinamide, and ethambutol, followed by 4 months of isoniazid and rifampicin (2HRZE/4HR). Patients were referred for antiretroviral therapy (ART) initiation if not already started, according to then-current national guidelines. Patients with drug resistant TB or in need of specialized TB treatment were not eligible, but were referred to an appropriate clinic. Socio-demographic data; prior episodes of TB; recent or current exposure to other anti-TB drugs; smoking and alcohol habits; occupational mining and dust exposure history; and antiretroviral treatment history were collected. Baseline patient measurements and investigations included: weight; height; chest x-ray; spot and/or overnight study sputum; CD4 count; viral load and full blood count. Sputum samples were collected from participants at baseline and then after initiation of drug therapy, at days 3, 7, 14, 28 and 35. In this analysis, data from day 28 (21 patients) and day 35 (84 patients) were combined, and designated as the “day 35” time point. For patients who missed time points due to loss to follow up, or for other reasons, earlier time points were included.

### Quantification of mycobacteria

All specimens were processed and analysed in research laboratories. Standard diagnostic tests were performed on decontaminated sputum samples according to the South African National Health Laboratory Service (NHLS) guidelines: smear microscopy by auramine O stain, Xpert and MGIT. In addition the number of colony forming units were determined on Middlebrook 7H11 agar medium supplemented with 0.5% OADC (oleic acid, albumin, dextrose, catalase, Becton Dickinson, South Africa) and documented as the logarithm of the colony forming units per millilitre (logCFU) [21], counting duplicate 10-fold serial dilutions series.

### Data analysis

This was an exploratory descriptive study and a sample of 118 patients was included, anticipating that baseline smear status of the entire group would be similarly represented. For each patient attending a study visit, all four semi-quantitative assays were performed on sputum and outputs were given as: auramine smear grade for microscopy, MTB grading and Ct values as supplied by standard Xpert documentation, TTP for MGIT and logCFU for viable counts. Continuous data were reported as medians and interquartile ranges for Xpert, MGIT and bacterial load. Negative results were documented for Xpert as a Ct of 40, for MGIT as a TTP of 42 days and for viable counts as logCFU = 0. For analysis of discrete data, positive results for microscopy were recorded according to auramine grading (AU): scanty (AUSC), one plus positive (AUP1+), two plus positive (AUP2+), or three plus positive (AUP3+); Xpert was given as MTB detected (MTBD) at a Ct of < 36; MGIT as TTP < 42 days and viable counts as logCFU > 0. Early bactericidal activity (EBA) is the fall in viable counts/ml sputum/day, calculated from colony forming units on solid medium at given time points. Data were analysed using GraphPad Prism 8. Comparison of continuous data was conducted by the Kruskal-Wallis method (non-parametric) or the students t-test (parametric). The difference in slope comparisons was estimated using linear mixed modelling.

## Results

### Patient demographics and clinical characteristics

From August 2013 to August 2014, we recruited 118 HIV-infected adults with pulmonary TB as evidenced by hard copy of a positive Xpert result taken routinely at a public sector clinic. At baseline, all patients were classified by the study smear result (Table S1): 41 (35%) were smear-negative and the other 77 (65%) smear-positive. Median age was 34 and 36 years, 19 and 22 kg / m^2^ for body mass index (BMI) and 82 and 141 cells / mm^3^ for CD4 count, in smear-negative and smear-positive patients, respectively. They differed significantly only by proportion of cavitation on chest x-ray: 28.6% of the smear-negative and 63% of smear-positive patients (Table 1).

**Table 1:** Baseline characteristics stratified by smear status

Variable	Overall	Smear positive	Smear negative	*P*-value
**Enrolment**	118	77	41	–
**Gender**				
Female (%)	60 (50.85)	43 (55.84)	17 (41.46)	0.1368
Male (%)	58 (49.15)	34 (44.16)	24 (58.54)	
**Median age (IQR) in years**	35.0 (31.0–42)	36.0 (31.0–42)	34.0 (29.5–43)	0.2595
**Median CD4 count (IQR)**	137 (11.0–274)	141 (0.00–272)	82.5 (20.0–274)	0.8428
**BMI**^a^				
Underweight (%)	31 (27.19)	22 (29.33)	9 (23.08)	0.6257
Normal (%)	66 (57.89)	41 (54.67)	25 (64.10)	
Overweight/Obese (%)	17 (14.91)	12 (16.00)	5 (12.82)	
**Median BMI (IQR)**	20.0 (18.3–22.7)	19.9 (18.0–22.7)	20.4 (18.6–22.8)	0.8018
**Cavities**^a^				
No (%)	36 (50.00)	16 (36.36)	20 (71.43)	0.0037
Yes (%)	36 (50.00)	28 (63.64)	8 (28.57)	
**Homeless within past year**^a^				
No (%)	81 (98.78)	51 (100.0)	30 (96.77)	–
Yes (%)	1 (1.22)	0 (0.00)	1 (3.23)	
**Not employed within the past 12 months**^a^				
No (%)	44 (53.66)	30 (58.82)	14 (45.16)	0.2290
Yes (%)	38 (46.34)	21 (41.18)	17 (54.84)	
**Alcohol use within the past year?**^a^				
No (%)	39 (47.56)	26 (50.98)	13 (41.94)	0.4265
Yes (%)	43 (52.44)	25 (49.02)	18 (58.06)	
**Did you smoke at least 2 cigarettes a week in the past month**				
No (%)	87 (73.73)	58 (75.32)	29 (70.73)	0.5893
Yes (%)	31 (26.27)	19 (24.68)	12 (29.27)	

*IQR* Interquartile range

^a^Totals may not be equal to the sample size as a result of missing values

### Accuracy of smear status at baseline

**Fig. 1:**
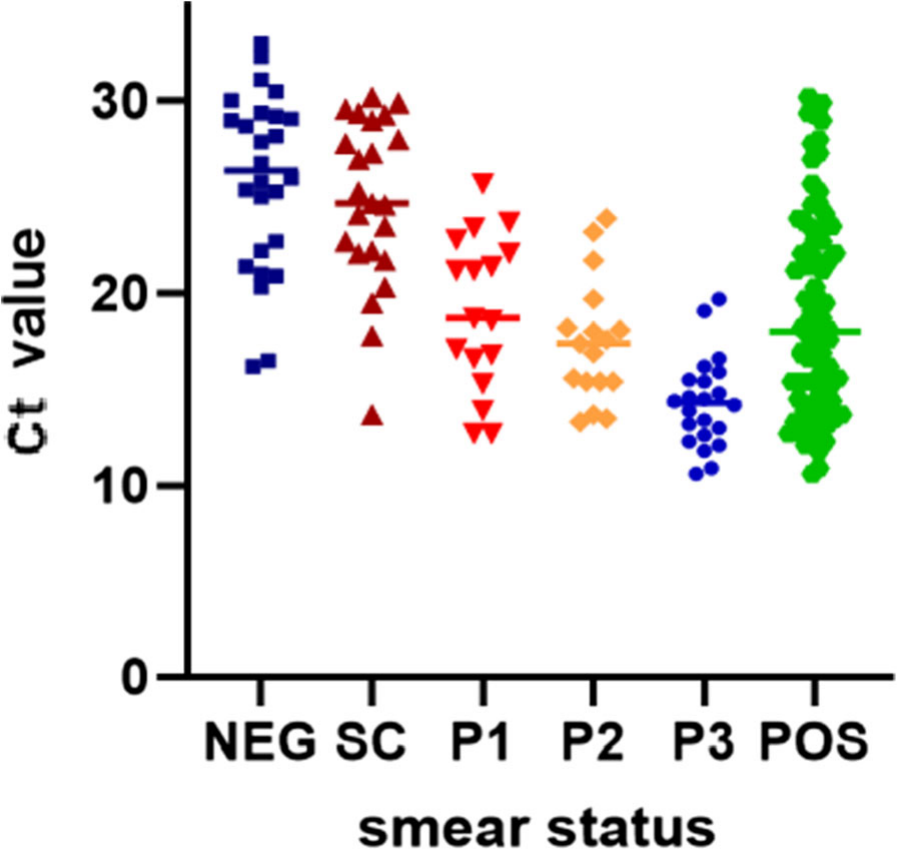
Comparison at enrolment of Xpert threshold values (Ct) against bacterial load as determined by smear microscopy status. Dark blue squares, AUNEG; dark red triangles, AUSC; red inverted triangles, AUP1+; orange diamonds, AUP2+; blue circles, AUP3+, green hexagons, AUPOS (AUSC, AUP1+, AUP2+ and AUP3+)

Overall, at baseline, higher smear status reflected lower Ct values (Fig. 1). However, of the 41 patients that were smear-negative (AUNEG), 27 (65.9%) were positive by Xpert diagnosis (Table S1); of these, the Ct values ranged between 16.2 and 33. In smear-positive patients (AUPOS) Ct values ranged between and 11.0 and 30.2. At baseline, 23 (85.2%) and 63 (96.9%) of smear-negative and smear-positive patients, respectively, were MGIT positive for *M. tuberculosis*. Of the culture negative samples, 14 (34.1%) also had a baseline negative Xpert result. Importantly, all participants, irrespective of smear status had at least one positive culture result during the study (MGIT and/or colony count culture).

### Response to therapy

**Fig. 2:**
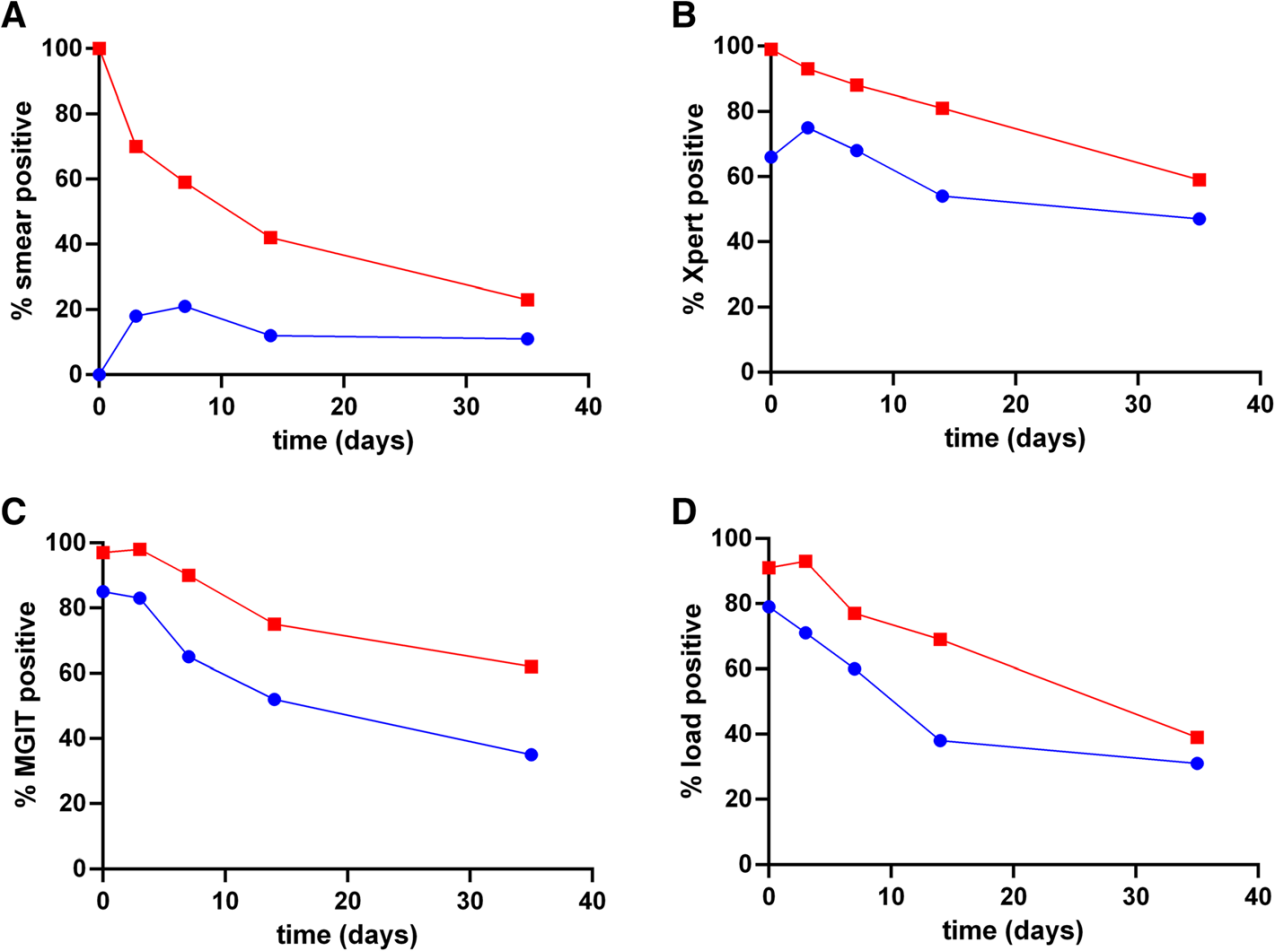
Percentage positivity for patient smear-positive (red curves) and smear-negative (blue curves) patient groups up to day 35. Results were based on **a** smear status, **b** Xpert, **c** MGIT and **d** viable counts

**Fig. 3:**
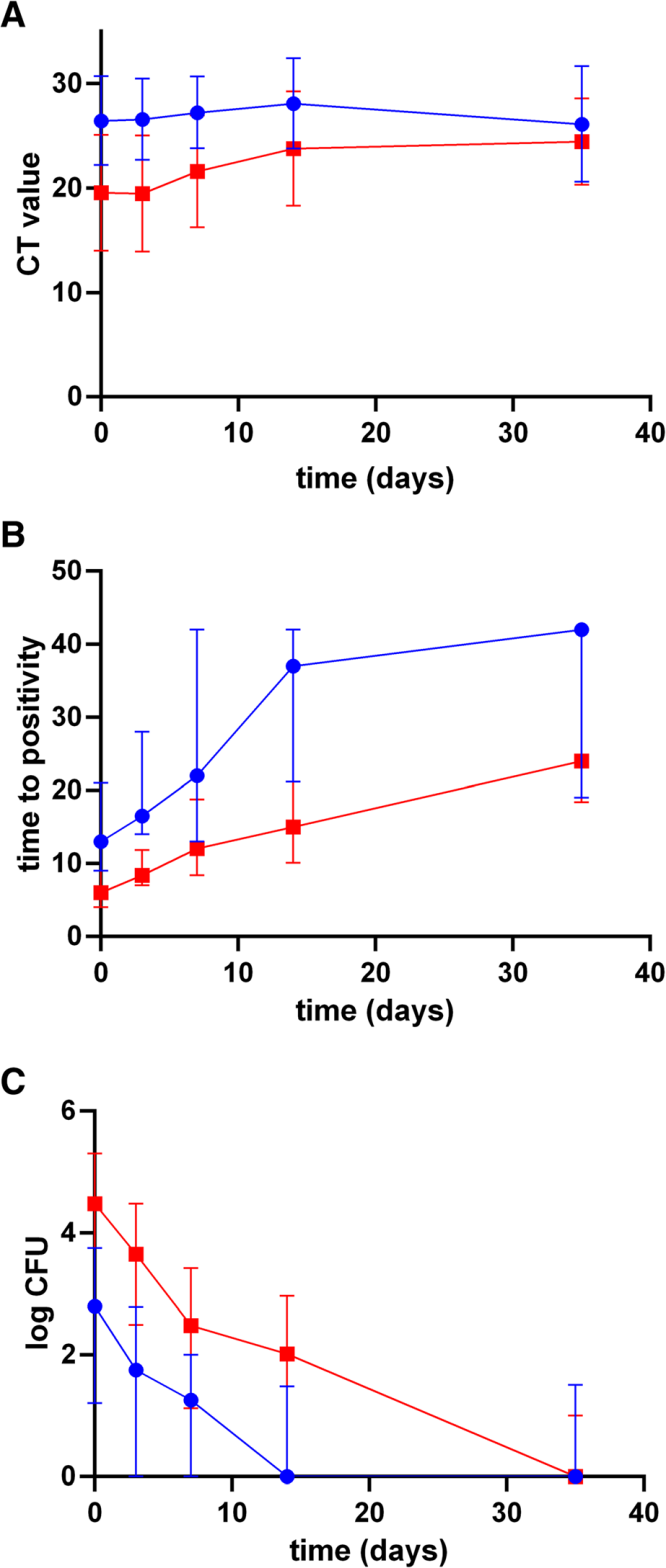
Semi-quantitative comparison between the smear-positive (red curves) and smear-negative (blue curves) patient groups. Change in bacterial load during treatment duration. Values are given as median ± interquartile range. **a** Xpert threshold values (Ct), **b** MGIT time to positivity (TTP) and **c** viable counts (logCFU). * *p* < 0.05 ** *p* < 0.005 *** *p* < 0.001 **** *p* < 0.0001

Standard four-drug TB treatment resulted in rapid reductions in all measures of bacillary load in both smear-negative and smear-positive groups (Fig. 2a – d). Moreover, at every time point, the four measures from the smear-positive patients had a higher percentage of positive results than from the smear-negative patients. Difference in slope by baseline smear status for the proportion that remained positive was statistically significant for all three measures (*p* < 0.001 on each comparison). By day 35 sputum specimens were positive in smear-negative and smear-positive patients, respectively in 11.1 and 28.6% by smear, 47.2 and 59.3% by Xpert, 35.5 and 61.5% by MGIT, and 22.6% and 38.7 by viable colony counts (Table S1). All median values reflected decreased bacillary load over time, with increasing Xpert Ct and MGIT TTP; and decreasing viable counts (Fig. 3a, b and c respectively, Table S2). A requirement for EBA studies is a serial quantifiable measure of mycobacterial load to determine response to drug therapy [22]. The EBA values for smear-negative and smear-positive groups were − 0.21 logCFU/ml/day and − 0.29 logCFU/ml/day, respectively until day 7. The bacterial load at baseline in the smear-negative patients is thus sufficient to allow for comparison of clearance upon drug treatment.

## Discussion

We compared a cohort of smear-negative and smear-positive PLH for their initial bacterial burden and early treatment responses. Severity of lung cavitation corresponded with baseline bacterial load, as expected [4]. While all measures of initial bacterial burden in the smear-positive was higher than the smear-negative patients, bacillary decline was able to be measured in both groups that were positive on subsequent measures. Our data suggest that, overall, 90% of HIV-infected adults diagnosed by Xpert alone, irrespective of smear status, would have a simultaneous positive MGIT culture result, and the majority of these could have serial measures of bacillary load whilst taking treatment.

Although most smear-negative patients were baseline culture positive (85.2% by MGIT and 79.2% by Xpert), this was lower than in smear-positive patients (96.9% by MGIT and 90.8% by Xpert); baseline bacterial load of smear-negative patients was 1.61 fold lower than that of the smear-positive group and bacteria were cleared in less than half the time in smear-negative patients, compared to smear-positive. Indeed, by day 14 MGIT TTP values approached 42 days in smear-negative patients. Our data also suggest that smear-negative TB patients may benefit from shortened treatment regimens [23, 24]. Moreover, serial bacterial counts in this early treatment period may be clinically relevant to rapidly identify patients not responding well to TB treatment. Xpert tests are increasingly available in clinical settings and could be used to quantify declines in bacillary load in practice, particularly in those who are smear-positive or have a low Ct value at baseline. Ct values of the smear-negative group did not change during treatment, because they reached the limit of detection (26.8 to 27.8, Fig. 3a). In the smear-positive group serial Ct values started lower and increased by day 14 (18.7 to 20.6), suggesting that at least in smear-positive patients or those with an initial low Ct value the Xpert assay has potential for rapidly identifying TB patients who are not responding to therapy. However, a substantial proportion (31.4%) of smear-negative patients had a negative Xpert test at baseline.

As a group, most smear-negative patients included in this study had sufficient viable bacteria that could be analysed meaningfully up to day 14 but one third of the smear-negative patients were also Xpert negative at baseline. MGIT tests have been reported to be at least equally as sensitive as colony counts [25–27] and we found at least 80% of smear-negative patients were culture positive at baseline.

There were several limitations to our study. This was a three-site study from one country; we did not document adherence of tuberculosis treatment or antiretroviral therapy; missing laboratory values and losses to follow up may have biased our results. The Xpert Ultra has largely replaced the assay we used to determine declines in bacterial load in this study. The sample size was small and we had fewer smear-negative patients in our analyses than we originally anticipated, and thus were unable to model the site effect. Moreover, apart from documenting the proportion of culture negative smear-negative PLH, we did not model sample size and cost implications of late exclusions of smear-negative TB patients from clinical trials who are subsequently found to be culture negative.

## Conclusion

Our data suggest that treatment responses in smear-negative HIV-infected TB patients whose initial positive diagnostic test was Xpert are measurable in most patients, suggesting that smear-negative PLH could be included in trials of novel TB therapies. Serial Xpert testing reflects treatment responses and could be a useful real-time measure of treatment response, but more research is required to ascertain if this holds true with the Xpert Ultra and to determine if serial measures could predict long term TB treatment outcomes. HIV-infected smear-negative patients can be included in clinical trials provided sample sizes are adjusted upwards for those that are eventually found to be culture negative.

## Supplementary Information

**Additional file 1: Supplementary Table S1.**

**Additional file 2: Supplementary Table S2.**

## Data Availability

The datasets used and/or analysed during the current study are available from the corresponding author on reasonable request.

## Abbreviations

TB: Tuberculosis
PLH: Persons living with HIV
ART: Antiretroviral therapy
Xpert: GeneXpert MTB/RIF
Ct: Cycle threshold
NAAT: Nucleic acid amplification technology
LOD: Limit of detection
CFU: Colony forming unit
MGIT: Mycobacterial growth indicator tubes
MTBD: MTB detected
TTP: Time to positivity
NHLS: National Health Laboratory Service
EBA: Early bactericidal activity
