# Supplementary material for: Comparing rates of mycobacterial clearance in sputum smear-negative and smear-positive adults living with HIV
Source: BMC Infect Dis. 2021 May 22;21:466. doi: 10.1186/s12879-021-06133-4 (PMC8141145; doi:10.1186/s12879-021-06133-4)
Supplement: Supplementary file 1 — Additional file 1: Supplementary Table S1. [file 12879_2021_6133_MOESM1_ESM.docx]

|  |  | **smear status** | | | **Xpert** | | | **MGIT** | | | **viable counts** | | |  |
| --- | --- | --- | --- | --- | --- | --- | --- | --- | --- | --- | --- | --- | --- | --- |
| **118 patients** | |  | | |  | | |  | | |  | | |  |
|  | **number of**  **patients** | **number of**  **read outs** | **AUPOS** | **Percent**  **Positive** | **number of**  **read outs** | **MTBD^a^** | **Percent**  **Positive** | **number of**  **read outs** | **TTP**  **<42 ^b^** | **Percent**  **Positive** | **number of**  **read outs** | **logCFU**  **>0 ^c^** | **Percent**  **Positive** |  |
| **Day 0** | 118 | 118 | 77 | 65.3 | 118 | 103 | 87.3 | 92 | 86 | 93.5 | 89 | 78 | 87.6 |  |
| **Day 3** | 104 | 87 | 43 | 49.4 | 104 | 90 | 86.5 | 87 | 81 | 93.1 | 81 | 69 | 85.2 |  |
| **Day 7** | 104 | 88 | 39 | 44.3 | 104 | 84 | 80.8 | 75 | 62 | 82.7 | 76 | 55 | 72.4 |  |
| **Day 14** | 100 | 86 | 26 | 30.2 | 100 | 71 | 71.0 | 79 | 53 | 67.1 | 73 | 43 | 58.9 |  |
| **Day 35** | 95 | 85 | 18 | 21.2 | 95 | 52 | 54.7 | 70 | 35 | 50.0 | 62 | 19 | 30.6 |  |
|  |  |  |  |  |  |  |  |  |  |  |  |  |  |  |
|  |  | **smear status** | | | **Xpert** | | | **MGIT** | | | **viable counts** | | |  |
| **smear negative**  **n = 41** | |  | | |  | | |  | | |  | | |  |
|  | **number of**  **patients** | **number of**  **read outs** | **AUPOS** | **Percent**  **Positive** | **number of**  **read outs** | **MTBD** | **Percent**  **Positive** | **number of**  **read outs** | **TTP**  **<42** | **Percent**  **Positive** | **number of**  **read outs** | **logCFU**  **>0** | **Percent**  **Positive** |  |
| **Day 0** | 41 | 41 | 0 | 0.0 | 41 | 27 | 65.9 | 27 | 23 | 85.2 | 24 | 19 | 79.2 |  |
| **Day 3** | 36 | 34 | 6 | 17.6 | 36 | 27 | 75.0 | 29 | 24 | 82.8 | 24 | 17 | 70.8 |  |
| **Day 7** | 37 | 34 | 7 | 20.6 | 37 | 25 | 67.6 | 23 | 15 | 65.2 | 20 | 12 | 60.0 |  |
| **Day 14** | 37 | 34 | 4 | 11.8 | 37 | 20 | 54.1 | 27 | 14 | 51.9 | 24 | 9 | 37.5 |  |
| **Day 35** | 36 | 36 | 4 | 11.1 | 36 | 17 | 47.2 | 31 | 11 | 35.5 | 31 | 7 | 22.6 |  |
|  |  |  |  |  |  |  |  |  |  |  |  |  |  |  |
|  |  | **smear status** | | | **Xpert** | | | **MGIT** | | | **viable counts** | | |  |
| **smear positive**  **n = 77** | |  | | |  | | |  | | |  | | |  |
|  | **number of**  **patients** | **number of**  **read outs** | **AUPOS** | **Percent**  **Positive** | **number of**  **read outs** | **MTBD** | **Percent**  **Positive** | **number of**  **read outs** | **TTP**  **<42** | **Percent**  **Positive** | **number of**  **read outs** | **logCFU**  **>0** | **Percent**  **Positive** |  |
| **Day 0** | 77 | 77 | 77 | 100.0 | 77 | 76 | 98.7 | 65 | 63 | 96.9 | 65 | 59 | 90.8 |  |
| **Day 3** | 68 | 53 | 37 | 69.8 | 68 | 63 | 92.6 | 58 | 57 | 98.3 | 57 | 52 | 91.2 |  |
| **Day 7** | 67 | 54 | 32 | 59.3 | 67 | 59 | 88.1 | 52 | 47 | 90.4 | 56 | 43 | 76.8 |  |
| **Day 14** | 63 | 52 | 22 | 42.3 | 63 | 51 | 81.0 | 52 | 39 | 75.0 | 49 | 34 | 69.4 |  |
| **Day 35** | 59 | 49 | 14 | 28.6 | 59 | 35 | 59.3 | 39 | 24 | 61.5 | 31 | 12 | 38.7 |  |
|  |  |  |  |  |  |  |  |  |  |  |  |  |  |  |

^a^ MTB detected according to standard Xpert output

^b^ growth detected before day 42

^c^ at least one viable colony
