# Supplementary material for: Comparing rates of mycobacterial clearance in sputum smear-negative and smear-positive adults living with HIV
Source: BMC Infect Dis. 2021 May 22;21:466. doi: 10.1186/s12879-021-06133-4 (PMC8141145; doi:10.1186/s12879-021-06133-4)
Supplement: Supplementary file 2 — Additional file 2: Supplementary Table S2. [file 12879_2021_6133_MOESM2_ESM.docx]

|  |  | |  |  |  |
| --- | --- | --- | --- | --- | --- |
|  | **smear negative group** | | **smear positive group** | |  |
|  |  |  |  |  |  |
|  | **number of**  **samples** | **Xpert**  **median Ct ± IQR** | **number of**  **samples** | **Xpert**  **median Ct ± IQR** | **p ^a^** |
| **Day 0** | 41 | 26.8 (23.9-29.3) | 77 | 18.7 (14.6-23.6) | <0.0001 |
| **Day 3** | 36 | 25.8 (23.9-28.9) | 68 | 18.1 (14.5-23.4) | <0.0001 |
| **Day 7** | 37 | 27.8 (25.8-28.6) | 67 | 20.6 (16.9-25.3) | <0.0001 |
| **Day 14** | 37 | 28.7 (25.6-30.5) | 63 | 24.3 (19.2-27.5) | 0.0012 |
| **Day 35** | 36 | 27.1 (21.1-29.7) | 59 | 23.9 (19.9-27.2) | 0.28 |
|  |  | |  |  |  |
|  |  | |  |  |  |
|  | **smear negative group** | | **smear positive group** | |  |
|  |  |  |  |  |  |
|  | **number of**  **samples** | **MGIT**  **median TTP ± IQR** | **number of**  **samples** | **MGIT**  **median TTP ± IQR** | **p** |
| **Day 0** | 27 | 13.0 (9.2-20.0) | 65 | 6.0 (4.0-9.0) | <0.0001 |
| **Day 3** | 29 | 16.5 (14.0-21.0) | 58 | 8.4 (7.0-11.5) | <0.0001 |
| **Day 7** | 23 | 22.0 (13.5-42.0) | 52 | 12.1 (8.8-18.3) | 0.0045 |
| **Day 14** | 27 | 37.0 (21.6-42.0) | 52 | 15.0 (10.0-36.0) | 0.0042 |
| **Day 35** | 31 | 42.0 (20.0-42.0) | 39 | 24.0 (18.6-42.0) | 0.18 |
|  |  |  |  |  |  |
|  |  |  |  |  |  |
|  | **smear negative group** | | **smear positive group** | |  |
|  |  |  |  |  |  |
|  | **number of**  **samples** | **viable counts**  **median logCFU ± IQR** | **number of**  **samples** | **viable counts**  **median logCFU ± IQR** | **p** |
| **Day 0** | 24 | 2.8 (1.3-3.4) | 65 | 4.5 (2.8-5.3) | 0.0016 |
| **Day 3** | 24 | 1.8 (0.0-2.7) | 57 | 3.7 (2.5-4.5) | <0.0001 |
| **Day 7** | 20 | 1.3 (0.0-1.9) | 56 | 2.5 (1.4-3.4) | 0.0084 |
| **Day 14** | 24 | 0.0 (0.0-1.5) | 49 | 2.0 (0.0-2.9) | 0.0014 |
| **Day 35** | 13 | 0.0 (0.0-0.5) | 31 | 0.0 (0.0-0.8) | 0.72 |
|  |  |  |  |  |  |

^a^ Kruskal-Wallis significance test
